# Supplementary material for: Liposomal Drug Delivery of Blumea lacera Leaf Extract: In-Vivo Hepatoprotective Effects
Source: Nanomaterials (Basel). 2022 Jun 30;12(13):2262. doi: 10.3390/nano12132262 (PMC9268469; doi:10.3390/nano12132262)
Supplement: Supplementary file 1 [file nanomaterials-12-02262-s001.zip › nanomaterials-1760127-supplementary.pdf]

nanomaterials MDPI

Supplementary S1: Normality test for all biomarker experiments

Liver SOD

Test for normal distribution

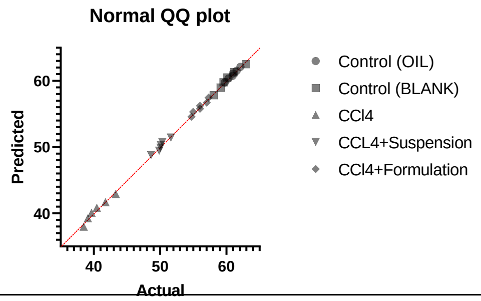

Kolmogorov-Smirnov test

KS distance	0.1627	0.1702	0.1725	0.2432	0.1719
P value	>0.1000	>0.1000	>0.1000	>0.1000	>0.1000
Passed normality test (alpha=0.05)?	Yes	Yes	Yes	Yes	Yes
P value summary	ns	ns	ns	ns	ns

Serum SOD

Liver GSH

Test for normal distribution

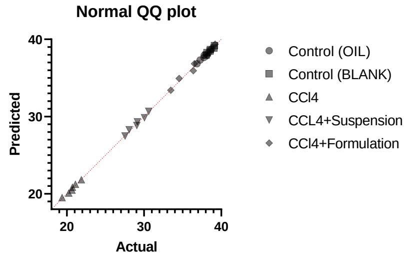

Kolmogorov-Smirnov test

KS distance	0.1657	0.1877	0.1683	0.1603	0.1648
P value	>0.1000	>0.1000	>0.1000	>0.1000	>0.1000
Passed normality test (alpha=0.05)?	Yes	Yes	Yes	Yes	Yes
P value summary	ns	ns	ns	ns	ns

Test for normal distribution

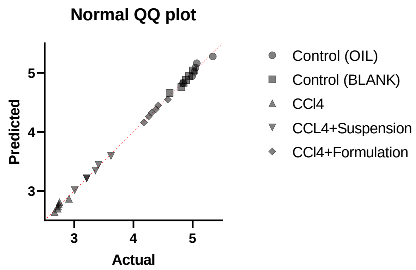

Kolmogorov-Smirnov test

KS distance	0.2926	0.2142	0.3496	0.1724	0.1538
P value	>0.1000	>0.1000	0.0209	>0.1000	>0.1000
Passed normality test (alpha=0.05)?	Yes	Yes	No	Yes	Yes
P value summary	ns	ns	*	ns	ns

Serum GSH

Test for normal distribution

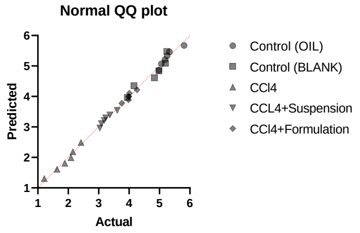

Kolmogorov-Smirnov test

KS distance	0.2502	0.2441	0.1727	0.2111	0.304
P value	>0.1000	>0.1000	>0.1000	>0.1000	0.0875
Passed normality test (alpha=0.05)?	Yes	Yes	Yes	Yes	Yes
P value summary	ns	ns	ns	ns	ns

Liver CAT

Test for normal distribution

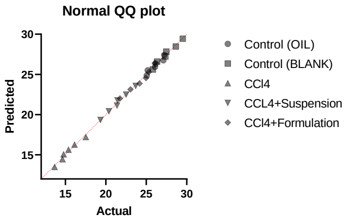

Kolmogorov-Smirnov test

KS distance	0.1893	0.1772	0.1658	0.171	0.1835
P value	>0.1000	>0.1000	>0.1000	>0.1000	>0.1000
Passed normality test (alpha=0.05)?	Yes	Yes	Yes	Yes	Yes
P value summary	ns	ns	ns	ns	ns

Serum CAT

Test for normal distribution

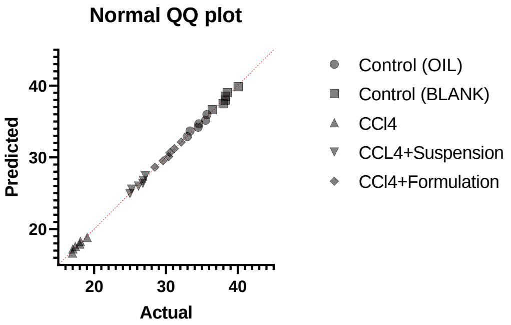

Kolmogorov-Smirnov test

KS distance	0.1787	0.2416	0.1837	0.2422	0.1661
P value	>0.1000	>0.1000	>0.1000	>0.1000	>0.1000
Passed normality test (alpha=0.05)?	Yes	Yes	Yes	Yes	Yes
P value summary	ns	ns	ns	ns	ns

Liver APOP

Test for normal distribution

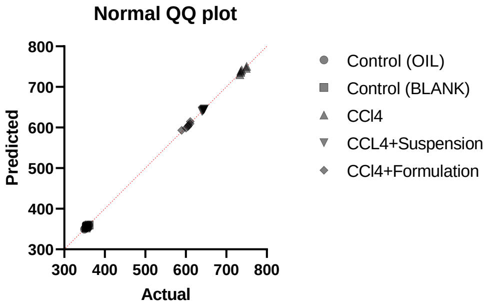

Kolmogorov-Smirnov test

KS distance	0.185	0.2244	0.2792	0.1769	0.2178
P value	>0.1000	>0.1000	>0.1000	>0.1000	>0.1000
Passed normality test (alpha=0.05)?	Yes	Yes	Yes	Yes	Yes
P value summary	ns	ns	ns	ns	ns

Serum APOP

Test for normal distribution

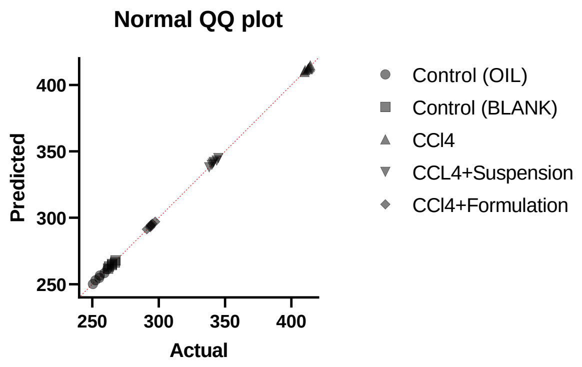

Kolmogorov-Smirnov test					
KS distance	0.1708	0.1731	0.1944	0.1622	0.2147
P value	>0.1000	>0.1000	>0.1000	>0.1000	>0.1000
Passed normality test (alpha=0.05)?	Yes	Yes	Yes	Yes	Yes
P value summary	ns	ns	ns	ns	ns

Liver NO

Test for normal distribution

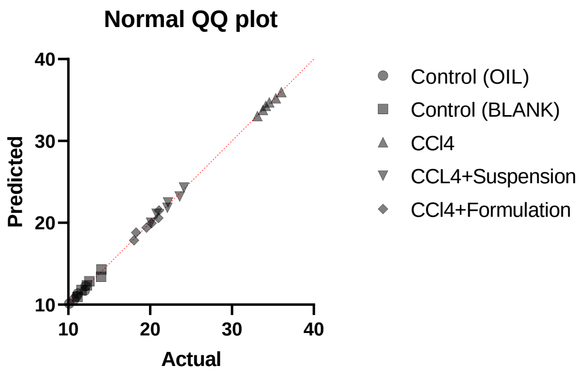

Kolmogorov-Smirnov test					
KS distance	0.192	0.2124	0.1404	0.1615	0.1922
P value	>0.1000	>0.1000	>0.1000	>0.1000	>0.1000
Passed normality test (alpha=0.05)?	Yes	Yes	Yes	Yes	Yes
P value summary	ns	ns	ns	ns	ns

Serum NO

Test for normal distribution

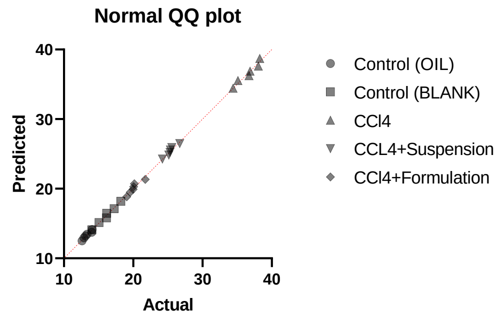

Kolmogorov-Smirnov test					
KS distance	0.2077	0.1644	0.2032	0.2456	0.3054
P value	>0.1000	>0.1000	>0.1000	>0.1000	0.0841
Passed normality test (alpha=0.05)?	Yes	Yes	Yes	Yes	Yes
P value summary	ns	ns	ns	ns	ns

<u>Liver MDA</u>

Test for normal distribution

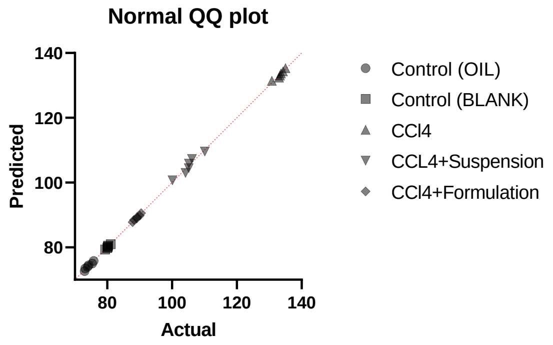

Kolmogorov-Smirnov test					
KS distance	0.1678	0.2669	0.2501	0.211	0.1601
P value	>0.1000	>0.1000	>0.1000	>0.1000	>0.1000
Passed normality test (alpha=0.05)?	Yes	Yes	Yes	Yes	Yes
P value summary	ns	ns	ns	ns	ns

<u>Serum MDA</u>

Test for normal distribution

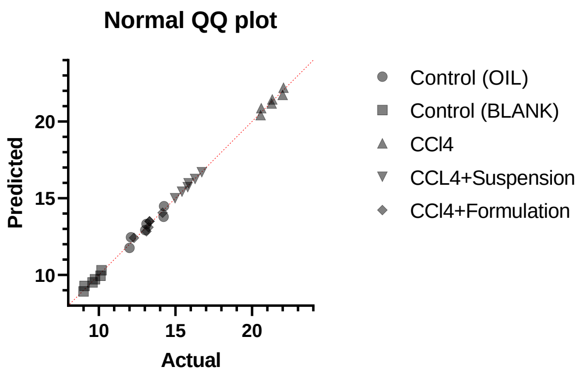

Kolmogorov-Smirnov test					
KS distance	0.203	0.1973	0.1938	0.1602	0.2876
P value	>0.1000	>0.1000	>0.1000	>0.1000	>0.1000
Passed normality test (alpha=0.05)?	Yes	Yes	Yes	Yes	Yes
P value summary	ns	ns	ns	ns	ns

MPO

Test for normal distribution

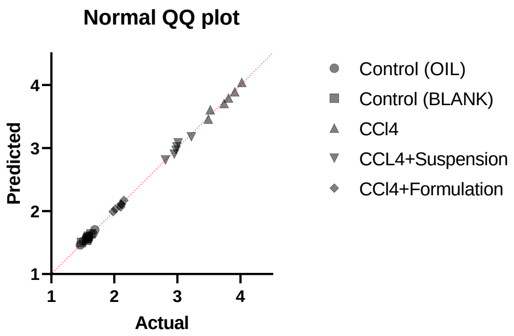

Kolmogorov-Smirnov test					
KS distance	0.159	0.2666	0.1937	0.2832	0.2986
P value	>0.1000	>0.1000	>0.1000	>0.1000	>0.1000
Passed normality test (alpha=0.05)?	Yes	Yes	Yes	Yes	Yes
P value summary	ns	ns	ns	ns	ns

ALT

Test for normal distribution

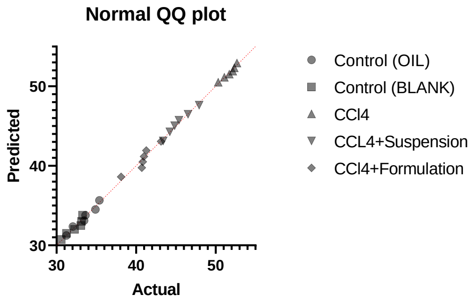

Kolmogorov-Smirnov test					
KS distance	0.1704	0.2629	0.1915	0.1683	0.2995
P value	>0.1000	>0.1000	>0.1000	>0.1000	0.0993
Passed normality test (alpha=0.05)?	Yes	Yes	Yes	Yes	Yes
P value summary	ns	ns	ns	ns	ns

<u>AST</u>

Test for normal distribution

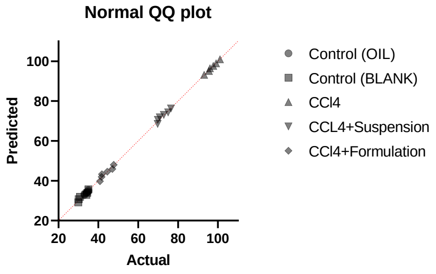

Kolmogorov-Smirnov test					
KS distance	0.2114	0.2518	0.1434	0.2234	0.2619
P value	>0.1000	>0.1000	>0.1000	>0.1000	>0.1000
Passed normality test (alpha=0.05)?	Yes	Yes	Yes	Yes	Yes
P value summary	ns	ns	ns	ns	ns

<u>ALP</u>

Test for normal distribution

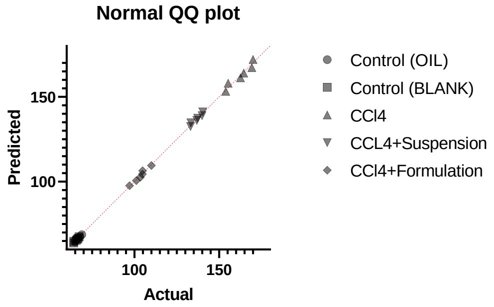

Kolmogorov-Smirnov test					
KS distance	0.1675	0.2076	0.1906	0.2033	0.2154
P value	>0.1000	>0.1000	>0.1000	>0.1000	>0.1000
Passed normality test (alpha=0.05)?	Yes	Yes	Yes	Yes	Yes
P value summary	ns	ns	ns	ns	ns

Supplementary S2: Non-parametric tests (Kruskal-Wallis test) for some biomarkers

**Table Analyzed**	**Serum SOD**
**Kruskal-Wallis test**	
P value	<0.0001
Exact or approximate P value?	Approximate
P value summary	****
Do the medians vary signif. (P < 0.05)?	Yes
Number of groups	5
Kruskal-Wallis statistic	24.07

**Table Analyzed**	**Liver NO**
**Kruskal-Wallis test**	
P value	<0.0001
Exact or approximate P value?	Approximate
P value summary	****
Do the medians vary signif. (P < 0.05)?	Yes
Number of groups	5
Kruskal-Wallis statistic	27.2

**Table Analyzed**	**Serum MDA**
**Kruskal-Wallis test**	
P value	<0.0001
Exact or approximate P value?	Approximate
P value summary	****
Do the medians vary signif. (P < 0.05)?	Yes
Number of groups	5
Kruskal-Wallis statistic	26.48

**Table Analyzed**	**ALT**
**Kruskal-Wallis test**	

P value	<0.0001
Exact or approximate P value?	Approxi-mate
P value summary	****
Do the medians vary signif. (P < 0.05)?	Yes
Number of groups	5
Kruskal-Wallis statistic	26.91

**Table Analyzed**	**MPO**
**Kruskal-Wallis test**	
P value	<0.0001
Exact or approximate P value?	Approxi-mate
P value summary	****
Do the medians vary signif. (P < 0.05)?	Yes
Number of groups	5
Kruskal-Wallis statistic	26.53
